# A *novel *heterozygous *missense *mutation in the UMOD gene responsible for Familial Juvenile Hyperuricemic Nephropathy

**DOI:** 10.1186/1471-2350-6-5

**Published:** 2005-01-27

**Authors:** Joaquim Calado, Augusta Gaspar, Carla Clemente, José Rueff

**Affiliations:** 1 Department of Genetics, Faculty of Medical Sciences, New University of Lisbon, Lisbon. Portugal; 2 Department of Nephrology, Santa Cruz Hospital, Lisbon. Portugal; 3 STAB GENÓMICA, Lisbon. Portugal

## Abstract

**Background:**

Familial Juvenile Hyperuricemic Nephropathy is an autosomal dominant nephropathy, characterized by decreased urate excretion and progressive interstitial nephritis. Mutations in the uromodulin coding UMOD gene have been found responsible for the disease in some families.

**Case presentation:**

We here describe a *novel *heterozygous p.K307T mutation in an affected female with hyperuricemia, renal cysts and renal failure. The proband's only son is also affected and the mutation was found to segregate with the disease.

**Conclusions:**

This mutation is the fourth reported in exon 5. Initial studies identified a mutation clustering in exon 4 and it has been recommended that sequencing this exon alone should be the first diagnostic test in patients with chronic interstitial nephritis with gout or hyperuricemia. However, regarding the increasing number of mutations being reported in exon 5, we now suggest that sequencing exon 5 should also be performed.

## Background

Familial Juvenile Hyperuricemic Nephropathy (FJHN) (McKusick 162000) is an autosomal dominant disorder characterized by hyperuricemia, decreased urinary excretion of urate and the development of progressive chronic interstitial nephritis. Renal impairment usually appears between 15 and 40 years of age, leading to end-stage renal disease (ESRD) within 10 to 20 years [1]. A candidate gene for FJHN has been positioned in 16p11.2-12, together with evidence for genetic heterogeneity [2,3]. The candidate gene was later found to map within the same genetic interval as MCKD2, a *locus* responsible for medullary cystic kidney disease, therefore suggesting that FJHN and MCKD2 are 2 facets of the same disease [1]. The marked thickening of tubular basement membranes observed in FJHN closely resembles the renal histological findings of the nephronophthisis-medullary cystic kidney disease complex (NPH-MCKD). Diseases of this group share the macroscopic feature of cyst development at the corticomedullary border of the kidney and the renal histological triad of tubular basement membrane disintegration, tubular atrophy with cyst development and interstitial cell infiltration with fibrosis [4]. Within this complex, clinical entities can be distinguished based on the mode of inheritance, the age of onset for ESRD and the presence of extra-renal involvement. For the recessive forms of the disease 4 different genes have been cloned. The NPHP1 gene [5,6] and NPHP4 [7] are responsible for juvenile forms of NPH, while NPHP2 [8] and NPHP3 [9] account for, respectively, the infantile and adolescent forms. MCKD, the autosomal dominant disorder that presents in early adulthood, is usually accompanied by the detection of corticomedullary cysts on imaging studies. MCKD1 maps to 1q21 and remains to be cloned, while MCKD2 has been positioned in 16p12. Mutations in the uromodulin/Tamm-Horsfall protein coding UMOD gene located within the critical interval of FJHN and MCKD2 at 16p11.2-12 were recently identified in FJHN and MCKD2 families [10], therefore providing definite evidence that MCKD and FJHN are allelic disorders. Meanwhile, a mutation cluster in exon 4 of UMOD was reported for both diseases [11,12].

We here describe a *novel *heterozygous *missense *mutation in affected individuals from a Portuguese FJHN family that also displays corticomedullary cysts on ultrasound examination. This mutation, c.920A→C, resides in exon 5 and is the fourth reported outside exon 4.

## Case presentation

### Case report

The proband is an affected female who was first evaluated at the age of 24, when she presented with a gout attack and hyperuricemia. At age 27 she was told having renal failure. However, a renal biopsy was not performed. At age 44, serum creatinine was 2.8 mg/dl and ultrasound imaging detected numerous renal cysts. Renal disease slowly progressed and the patient reached ESRD when she was 49 years old. Her father died at the age of 55 from ESRD and suffered from hyperuricemia and gout. The proband's only son is also affected. At the age of 18 years he had a gout attack. On the initial evaluation, serum uric acid was 15 mg/dl and serum creatinine 1.6 mg/dl. Renal cysts were also detected on ultrasound examination.

### Mutation analysis

Informed consent was obtained from tested individuals. Genomic DNA was isolated from peripheral blood leucocytes and the coding region of the UMOD gene was screened for mutations by direct sequencing of PCR products. We used a set of primers previously described [10] except in exons 4 and 5, for which different additional internal sequencing primer were designed based upon sequences from GenBank (accession numbers NT_024776.6 and M17778).

### Results

**Figure 1:**
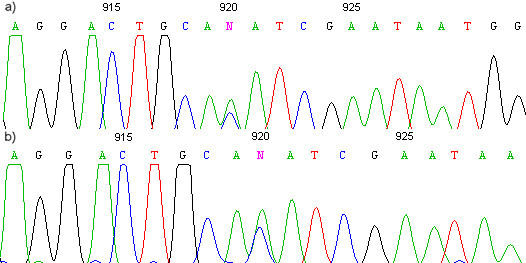
**a) **Sequence of the index patient with exon 5 c.920A→C heterozygous *missense *mutation. **b) **The same mutation sequenced with a reverse primer.

A *novel *heterozygous *missense *mutation, c.920A→C, was detected (Figure 1) and found to segregate with the disease in this family. The mutation results in a Lys to Thr at position 307. This mutation was not detected in any of the 100 control chromosomes tested. In fact, no polymorphism affecting the translation of uromodulin was detected in 100 control chromosomes in a previous report [10] and we are, therefore, excluding the possibility of this allele being a mere polymorphism. In addition, the affected mother was found to be homozygous for the T allele in the common T to C transition synonymous polymorphism at codon C174.

### Discussion

The UMOD gene has 12 exons and codes for the 640 amino-acid uromodulin, a glycsoyl phosphatidylinositol (GPI) anchored protein that accounts for the primary structure of the 85-kD Tamm-Horsfall glycoprotein (THP). THP is the most abundant protein in the urine and, in normal kidney, uromodulin expression is restricted to the thick ascending limb (TAL) and distal convoluted tubule. Urinary excretion occurs by proteolytic cleavage of the GPI counterpart at the luminal surface of TAL. It has been suggested that one of THP major role is of an urinary anti-adherence factor preventing type 1 fimbriated E coli from binding to the urothelial receptors [13].

The majority of the mutations so far published are clustered in exon 4, between codons 52 and 282, and most are *missense *mutations affecting cysteine residues (Table 1). Exon 4 contains 3 calcium binding epidermal growth factor (cbEGF)-like domains, between residues 31 and 148. A fourth potential cbEGF-like domain extends from amino-acids 281–336, throughout exon 5. They contain 6 conserved cysteine residues responsible for the protein's tertiary structure, as a result of intramolecular disulfide bonding. It has been hypothesized that protein misfolding, consequence of mutations in these cbEGF-like domains, may affect uromodulin intracellular trafficking and lead to cellular protein accumulation and apoptosis [14]. The release of cells debris and uromodulin aggregates in the interstitium could stimulate an inflammatory response and, in addition, be responsible for tubular obstruction and medullary cyst formation. It has been proposed that hyperuricemia in these patients is secondary to a reduced TAL sodium reabsorption with volume contraction and a compensatory increase in proximal urate reabsorption. The role of hyperuricemia in the chronic interstitial nephritis remains to be clarified, since the deposition of sodium urate crystals in the medullary interstitium does not occur in these patients. The mutation c.920A→C here reported, replaces the basic amino-acid Lys for the uncharged polar Thr at position 307 (p.K307T), being the fourth mutation described in exon 5. The affected residue locates within the fourth cbEGF-like domain and is immediately preceded by a highly conserved cysteine residue.

## Conclusions

It has been referred that exon 4 sequencing should become the first diagnostic test in patients with chronic interstitial nephritis with gout or hyperuricemia, even in the absence of a family history [11]. In view of the increasing number of mutations in exon 5 being identified, we now recommend that exon 5 should be included in the initial sequencing effort, since otherwise nearly 12% of UMOD mutations can be missed.

## Competing interests

The author(s) declare that they have no competing interests.

## Authors' contributions

JC was responsible for the study design and drafted the manuscript. JC and CC carried out the molecular analysis. AG collected the clinical data. JR participated in the study design.

## Pre-publication history

The pre-publication history for this paper can be accessed here:



## Figures and Tables

**Table 1:** UMOD mutations reported in the literature.

Mutation	exon	reference
c.156T→G; p.C52W	4	[15]
c.176A→C; p.D59A	4	[11]
c.230G→A; p.C77Y	4	[16]
c.278_289del/insCCGGCTCCT; p.V93_G97del/insAASC	4	[12]
c.307G→T; p.G103C	4	[10]
c.334T→C; p.C112R	4	[11]
c.376T→C; p.C126R	4	[16]
c.383A→G; p.N128S	4	[16]
c.403T→A; p.C135S	4	[15]
c.443G→A; p.C148Y	4	[10]
c.444T→G; p.C148W	4	[14]
c.449G→C; p.C150S	4	[14]
c.509G→A; p.C170Y	4	[11]
c.529_555del; p.H177_R185del	4	[10]
c.553C→G; p.R185G	4	[18]
c.553C→A; p.R185S	4	[11]
c.563_661del; p.E188_L221del	4	[11]
c.584G→T; p.C195F	4	[15]
c.605G→C; p.W202S	4	[15]
c.610C→G; p.R204G	4	[11]
c.649T→C; p.C217R	4	[10]
c.649T→G; p.C217G	4	[11]
c.665G→C; p.R222P	4	[11]
c.668G→A; p.C223Y	4	[17]
c.674C→T; p.T225M	4	[11]
c.674C→A; p.T225K	4	[12]
c.707C→T; p.P236L	4	[15]
c.744C→G; p.C248W	4	[12]
c.764G→A; p.C255Y	4	[16]
c.844T→C; p.C282R	4	[11]
c.898T→G; p.C300G	5	[16]
c.920A→C; p.K307T	5	present report
c.943T→C; p.C315R	5	[14]
c.950G→A; p.C317Y	5	[14]
